# CD157^+^ vascular endothelial cells derived from human-induced pluripotent stem cells have high angiogenic potential

**DOI:** 10.1186/s41232-025-00379-0

**Published:** 2025-05-14

**Authors:** Ami Takii, Yukika Tanabe, Wenting Li, Hiroki Shiomi, Akane Inoue, Fumitaka Muramatsu, Weizhen Jia, Nobuyuki Takakura

**Affiliations:** 1 Department of Signal Transduction, Research Institute for Microbial Diseases, The University of Osaka, Suita, Osaka Japan; 2 World Premier Institute Immunology Frontier Research Center, The University of Osaka, Osaka, Japan; 3 Integrated Frontier Research for Medical Science Division, Institute for Open and Transdisciplinary Research Initiatives (OTRI), The University of Osaka, Osaka, Japan; 4 Center for Infectious Disease Education and Research, The University of Osaka, Osaka, Japan

**Keywords:** Angiogenesis, CD157, Endothelial cells, Human iPSC, Stem cells

## Abstract

**Background:**

We previously reported that a vascular endothelial stem cell population resides in pre-existing blood vessels in mice and may contribute to vascular endothelial cells in liver injury or hind limb ischemia models in the long-term. However, whether such stem cells exist in humans and can differentiate specifically into vascular endothelial cells have not been determined. We hypothesized that CD157^+^ vascular endothelial cells in humans may also possess high angiogenic potential.

**Methods:**

First, human-derived induced pluripotent stem cells were differentiated into vascular endothelial cells and the expression of CD157 was monitored during the differentiation process. We found that CD157 emerged 11 days after the induction of differentiation, peaked at 14 days, and then declined by 24 days. We also evaluated blood vessel formation by 14- and 24-day-old vascular endothelial cells.

**Results:**

It was found that 14-day-old cells, when CD157 expression was at its peak, formed more blood vessels than 24-day-old cells.

**Conclusion:**

These results suggest that vascular endothelial cells expressing CD157 have high angiogenic potential and may exist as vascular endothelial stem cells.

**Supplementary Information:**

The online version contains supplementary material available at 10.1186/s41232-025-00379-0.

## Background

Blood vessels play a crucial role in supplying oxygen and nutrients to organs and tissues throughout the body and in facilitating the delivery of immune cells to damaged areas. Endothelial cells, located in the intraluminal surface layer of blood vessels, secrete cytokines with angiocrine functions that play a significant role in maintaining organ homeostasis and tissue regeneration, and that promote angiogenesis. Additionally, endothelial cells are closely associated with the regulation of processes such as vascular leakage [1].

Angiogenesis refers to the formation of new blood vessels from pre-existing ones and is essential in processes occurring in wound healing and pregnancy. During angiogenesis, endothelial cells extend filopodia in response to ischemic conditions and environmental cues, leading to the emergence of tip cells that guide the direction of vascular extension. Subsequently, highly proliferative stalk cells appear, contributing to the elongation of blood vessel branches. Finally, phalanx cells emerge inducing the maturation of blood vessels through processes such as pericyte adhesion and ultimately forming new blood vessels [2, 3]. Due to the diversity of endothelial cells it has been suggested that these cells include a population with stem cell-like properties referred to as endothelial stem cells. Such cells have the potential to differentiate into various types of endothelial cells. Endothelial stem cells, positioned at the apex of the differentiation hierarchy, exhibit robust angiogenic potential by being highly proliferative. Consequently, transplantation of endothelial stem cells is anticipated to yield significant improvements in ischemia, and facilitate organ and tissue regeneration.

Endothelial stem cells are a subset of somatic stem cells, which are present throughout the body, possessing the ability to self-renew and differentiate into terminally differentiated vascular endothelial cells [4]. Somatic stem cells are restricted to differentiating into cells associated with their respective tissues, such as liver stem cells differentiating into liver cells and hematopoietic stem cells into blood cell lineages. Somatic stem cell transplantation, exemplified by hematopoietic stem cell transplantation for the treatment of leukemia, is relatively safe due to the low incidence of cancer.

We previously identified endothelial cells showing stem cell characteristics in mice; these expressed CD157 known as BST-1 [5, 6]. Using a side population (SP) method based on the drug-efflux property of stem cells [7], we isolated SP cells that expelled Hoechst DNA dye, resulting in Hoechst blue- and Hoechst red-negative endothelial cells [8]. Subsequent microarray analysis revealed high expression of CD157 in these SP cells. Transplantation of CD157^+^ cells isolated from the mouse liver demonstrated their persistent ability to construct blood vessels in regions with hepatic vascular disorder or ischemia in the lower limbs. Moreover, an improvement in bleeding abnormalities was observed in hemophilia A mice by transplanting CD157^+^ endothelial cells derived from wild-type mice. These findings highlight the potential clinical utility of endothelial stem cell transplantation for vascular and ischemic diseases.

However, for the clinical application of endothelial stem cells in humans, it is necessary to demonstrate the presence of human endothelial stem cells and whether CD157 expression marks the presence of endothelial stem cell-like cells. Here, we induced the differentiation of human induced pluripotent stem cells (iPSCs) into endothelial cells and verified whether CD157^+^ cells appeared, as found for mice. We also investigated stem cell-like properties with high angiogenic or transplantable potential.

Human iPSCs can differentiate into most cell types in our body [9]. Therefore, they have been applied to clinical settings such as cardiac [10] and corneal epithelial cell [11] sheets. Moreover, endothelial cells from human iPSCs have contributed to vascular formation in mice, leading to improvements in hemostatic abnormalities in hemophilia A mice [12].

For the experimental use of human endothelial cells, human umbilical vein endothelial cells (HUVECs) [13] are primarily employed; however, most such cells are CD157-negative and not transplantable. While endothelial cells can be obtained from various tissues, using human tissues poses challenges in terms of cost, ethical concerns, and difficulty in cultivation methods. For these reasons, we aimed to mimic vascular differentiation using human iPSCs and conduct a functional analysis of human endothelial stem cells. In this study, we investigated the functionality of human CD157^+^ endothelial cells using RNA sequencing analysis, a tube formation assay, and in vivo transplantation analysis.

## Methods

### Mice

NOD.Cg-*Prkdc *^*scid*^* Il2rg *^*tm1 Wjl*^*/SzJ* female mice (8-weeks-old) were purchased from The Jackson Laboratory Japan, Inc. (Kanagawa, Japan). All experimental procedures in this study were approved by the University of Osaka Committee for animal and recombinant DNA experiments. Mice were handled and maintained according to University of Osaka guidelines for animal experimentation.

### Cell culture

UTA-SF2-2 (JCRB Cell Bank, Osaka, Japan) human iPSCs were maintained on iMatrix-511™ (Nippi, Tokyo, Japan) substrate in StemFit® AK02 N (Ajinomoto, Tokyo, Japan) medium supplemented with 10 µM CultureSure®Y-27632 (Wako, Settsu, Japan). HUVECs were maintained in Humedia EG2 (KURABO, Osaka, Japan). These cells were passaged less than seven times by differentiation induction.

### Induction of differentiation of human iPSCs into endothelial cells

**Fig. 1:**
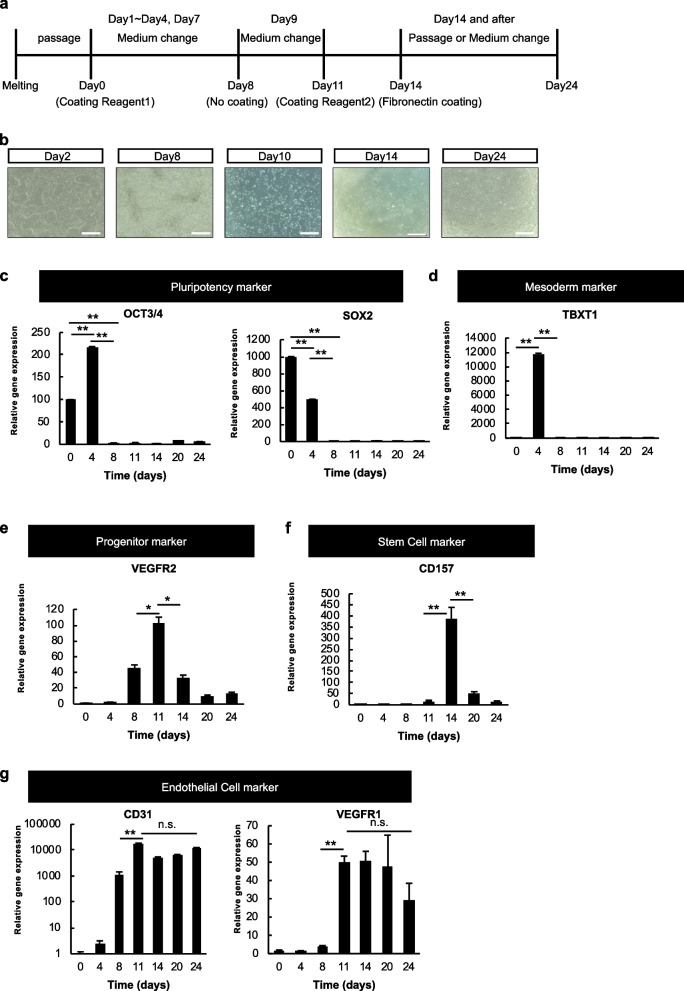
Induction of differentiation of human iPSCs into vascular endothelial cells. **a** Outline of the protocol for the induction of differentiation of human iPSCs to vascular endothelial cells. The induction of differentiation was performed according to a protocol using a MiraCell® iPS Cell to Endothelial Cell Differentiation Kit (TaKaRa). **b** Changes in cell morphology during the induction of differentiation: an iPSC-like morphology was observed on day 2, but a cobblestone-like morphology was seen in endothelium from day 14. Scale bar = 500 μm. **c**–**g** Relative gene expression levels of pluripotent stem cell markers (*OCT3*/4, *SOX2*) (**c**), mesoderm markers (*TBXT1*; Brachyury) (**d**), endothelial progenitor cell markers (*VEGFR2*) (**e**), endothelial stem cell markers (*CD157*) (**f**), endothelial cell markers (*CD31*, *VEGFR1*) (**g**) on days 0, 4, 8, 11, 14, 20, and 24 quantified by reverse transcription (RT)-PCR (*P < 0.05, **P < 0.01, n.s. not significant)

For the induction of differentiation into endothelial cells, a MiraCell® iPS Cell to Endothelial Cell Differentiation Kit (TaKaRa, Otsu, Japan) was utilized. Initially, human iPSCs were passaged more than two times after thawing frozen cells. We then induced differentiation of iPSCs into endothelial cells according to the instructions of this kit. In a brief overview of the induction of differentiation process: On day 0, human iPSCs were seeded on coating reagent 1 and on day 1 the medium was then changed. Additionally, on day 2, medium containing coating reagent 1 was added, and from day 3 onwards, supplements for the induction of differentiation were added. On day 8, passaging was carried out without the use of coating reagents. During passaging on day 11, coating reagent 2 was used. For culture after day 14, MiraCell® EC Culture Medium (TaKaRa) and fibronectin (Sigma–Aldrich, Tokyo, Japan) were used as a medium and coating reagent, respectively, according to the manufacturers’ instructions (Fig. 1a). Cells were collected on days 4, 8, 9, 11, 14, 20, and 24 during the induction of differentiation for quantitative reverse transcription (qRT)-PCR and flow cytometric analysis.

### Quantitative reverse-transcription PCR

RNA was extracted using RNeasy Mini Kits (Qiagen, Hilden, Germany) and cDNA was generated using reverse transcriptase from an EsScript RT Reagent Kit (TaKaRa). Quantitative reverse transcription-PCR was performed with a LightCycler 96 System (Roche, Basel, Switzerland) using a SYBR Premix Ex Taq II kit (TaKaRa) in accordance with the manufacturer’s instructions. Quantitative assessment of gene expression was performed using a standard ⊿Ct method applying *GAPDH* as a single internal control. The oligonucleotide primers used for targeted detections are listed in Supplementary Table S1.

### Flow cytometric analysis

Endothelial cells derived from iPSCs (iPSC-ECs) were washed with phosphate buffered saline (PBS), mixed with Accumax (Innovate Cell Technologies, Inc., San Diego, CA, USA), and the mixture then incubated for 4 min to detach cells. Cell surface antigen staining was performed with anti-CD31 (Clone WM59, BD Bioscience, San Jose, CA, USA), anti-CD45 (Clone 2D1, BD Bioscience), and anti-CD157 (Clone RF3, MBL International Woburn, MA, USA) antibodies. Stained cells were analyzed by a BD LSRFortessa™ X-20 flow cytometer (BD Bioscience) and data was analyzed using FlowJo Software (Treestar Software, San Carlos, CA, USA).

### Fluorescence immunostaining

After washing iPSC-ECs with PBS, 4% paraformaldehyde was added and cells incubated at room temperature for 10 min. Subsequently, PBS with 0.5% Tween-20 (0.5% PBST) was added for 5 min at room temperature for permeabilization, followed by a PBS wash. Blocking was performed by incubating the cells in Blocking Buffer (5% normal goat serum, 1% bovine serum albumin 2% skim milk/PBS) at room temperature for 1 h. After blocking, primary antibodies were added to the Blocking Buffer at a 1/100 dilution and left overnight at 4 °C. The next day, cells were washed three times with PBST. Secondary antibodies were added to the Blocking Buffer at a 1/300 dilution and incubated at room temperature for 2 h. After removal, fluorescein isothiocyanate conjugated anti-CD157 antibody was added to the Blocking Buffer at a 1/100 dilution and left overnight at 4 °C. The following day, cells were washed six times with PBST and PBS containing 4″,6-diamino-2-phenylindole (DAPI) at a 1/1000 dilution was added for 5 min. Finally, cells were washed with PBST and observed using a fluorescence microscope (FLUOVIEW FV3000, Olympus, Tokyo, Japan).

### RNA sequence analysis

iPSC-ECs (5 × 10^5^) from days 11, 14, and 24 cultures were dissolved using TRIzol (Thermo Fisher Scientific, Waltham, MA, USA). Total RNA was extracted using a miRNeasy Micro kit (Qiagen) following the manufacturer’s protocol. A library was prepared using a TruSeq stranded mRNA Library Prep kit (Illumina, San Diego, CA, USA) according to the provided instructions. RNA sequencing was carried out on a NovaSeq 6000 sequencer (Illumina) in 101-base single-read mode and raw counts were calculated using featureCounts v2.0.0. The fragments per kilobase of exon per million mapped fragments (FPKMs) were determined using Cuffdiff v2.2.1. The obtained FPKM files were used for inter-sample gene expression analysis using iDEP.96.

### Tube formation and Ac-LDL uptake assays

For gel preparation, 50 µL of growth factor reduced–Matrigel (Corning, NY, USA) was dispensed into each well of a 96-well plate and incubated at 37 °C in 5% CO₂ for 30 min. iPSC-ECs were suspended in MiraCell® EC Culture Medium supplemented with 80 ng/mL of vascular endothelial growth factor (VEGF; PeproTech Inc., Rocky Hill, NJ, USA), seeded at a density of 4 × 10^4^ or 8 × 10^4^ cells/well, and cultured overnight. Alexa Fluor™ 594-acetylated low density lipoprotein (Ac-LDL; Life Technologies, Carlsbad, CA, USA) was added to the medium 5 h before each observation; the medium was replaced immediately before each observation. For the observation of tube formation and fluorescence imaging, an inverted microscope (Dmi8; Leica, Nussloch, Germany) was utilized.

### Transplantation into mice with hind limb ischemia

A hind limb ischemia mouse model was set up as described previously [14]. In brief, the proximal parts of the right femoral artery and vein, and the distal parts of the saphenous artery and vein, were occluded and resected in NOD.Cg-*Prkdc *^*scid*^* Il2rg *^*tm1 Wjl*^*/SzJ* mice (8-weeks-old). CD157^+^ vascular endothelial cells were isolated from day 14 iPSC-ECs and CD157^−^ vascular endothelial cells were isolated from day 24 iPSC-ECs by flow cytometry using an Aria cell sorter (BD Bioscience). These cells were suspended in Mira Cell EC Culture Medium (TaKaRa, Japan) supplemented with 100 ng/mL VEGF. After ischemia was induced as above, 10 × 10^5^ CD157^+^ or CD157^−^ endothelial cells were transplanted into each hind limb muscle. After 2 weeks of transplantation, hind limb muscles were dissected from each leg. A single cell suspension was made [14], and cells were analyzed by flow cytometry (BD LSRFortessa™ X-20 flow cytometer; BD Bioscience). Moreover, the engraftment ability of human CD31^+^ endothelial cells was analyzed by immunofluorescence staining. The antibodies used were human CD31, mouse CD31 (1 st: clone 2H8, Sigma-Aldrich, USA; 2nd: Cy3 AffiniPure Goat Anti-Armenian Hamster IgG (H + L), USA), and TO-PRO-3 (Thermo Fisher Scientific, USA).

### Laser doppler blood flow imaging

Blood flow improvement in the lower limb was assessed using a laser doppler blood flow (LDBF) analyzer (Moor LDI2-IR; Moor Instruments). Two days after surgery, iPSC-ECs (1 × 10^5^ cells in 50 µL PBS) were transplanted into the biceps femoris muscle of the thigh. LDBF analysis was performed on the gastrocnemius muscle or the foot of the ischemic limb immediately before surgery and on postoperative days 4, 7, 10, and 14.

## Results

### Induction of differentiation of human iPSCs into endothelial cells

Upon examining the morphology of cells during the induction of differentiation, flattened colonies characteristic of primed human iPSCs were observed on day 2. On day 8, the flat colonies disintegrated and fibroblast-like cells began to appear in increased numbers. On day 10, spherical cells were present. On day 14, a cobblestone-like morphology commonly found in colonies of endothelial cells was evident. Although the morphology was maintained beyond day 14, a relatively enlarged cell size was observed from day 24 onward (Fig. 1b).

To analyze the proper execution of the induction of differentiation, we investigated relative gene expression levels of differentiation markers using qRT-PCR. The four Yamanaka factors (OCT3/4, SOX2, KLF4, MYC) are transcription factors that induce pluripotency and are typically expressed in undifferentiated cells. High expression of OCT3/4 and SOX2 was observed at stages close to undifferentiated states, such as days 0 and 4 (Fig. 1c). The mesodermal marker, TBXT1 (Brachyury), showed an increase in expression on day 4 (Fig. 1d). The expression of vascular endothelial growth factor receptor (VEGFR)2, expressed in mesoderm and endothelial cells, began on day 4, peaked on day 11, and gradually decreased thereafter (Fig. 1e). *CD157* is a gene previously found to be expressed in mouse endothelial stem cells. This gene exhibited a significant increase on day 14 during the induction of differentiation from human iPSCs to endothelial cells, followed by a decrease by day 20 (Fig. 1f). The endothelial cell markers, CD31 and VEGFR1, showed high expression on days 8 and 11, respectively, with CD31 maintaining its expression thereafter. In contrast, VEGFR1 exhibited decreased expression on day 24 (Fig. 1g). These results indicate the successful induction of differentiation and suggest the complete maturation of endothelial cells by day 24.

### Expression analysis of CD31 and CD157 during differentiation stages

To investigate how expression levels of CD31 and CD157 change during differentiation, expression analysis of CD31 and CD157 was conducted at different stages of differentiation using flow cytometry and fluorescence immunostaining. CD31 is a surface antigen expressed in some blood cells and endothelial cells, while CD45 is a surface antigen expressed exclusively in blood cells.

**Fig. 2:**
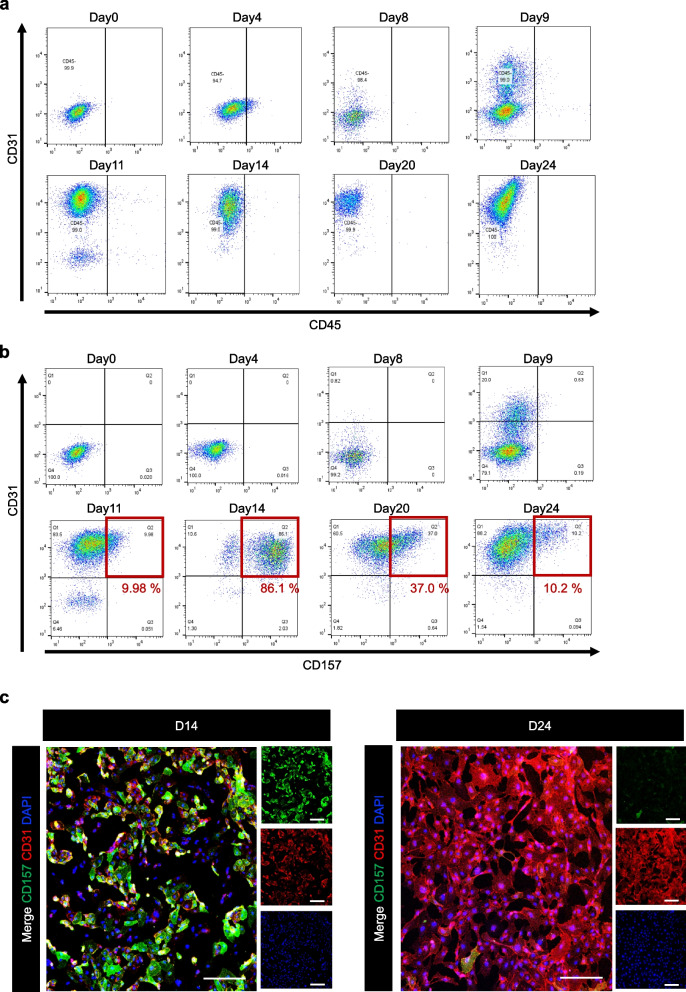
Analysis of CD31 and CD157 expression during the differentiation induction phase. **a** Flow cytometric analysis using CD31 (vertical axis) and CD45 (horizontal axis) antibodies during the differentiation induction phase from human iPSCs to vascular endothelial cells. **b** Flow cytometric analysis using CD31 (vertical axis) and CD157 (horizontal axis) antibodies. **c** Fluorescent immunostaining with antibodies for CD31 and CD157 on day 14 (left) and day 24 (right): green for CD157, red for CD31, and blue for DAPI; Scale bar = 250 μm

In flow cytometry analysis, endothelial cells were initially gated from the CD31^+^/CD45^−^ population, and within that cell population the percentage of CD157^+^ cells was explored. As a result, CD31 expression gradually increased from day 9 and maintained high expression thereafter. In contrast, CD45 expression remained low at all differentiation stages, suggesting the absence of cells from the blood cell lineage during the induction of differentiation in this method (Fig. 2a). Regarding CD157 expression, it was 9.98% on day 11, increased to 86.1% on day 14, gradually decreased from around day 20, and reached approximately 10% on day 24. This level was maintained even after prolonged cultivation post–day 24 (Fig. 2b). These results suggest that the expression of CD157 during the induction of differentiation from human iPSCs to endothelial cells is transient, with expression being sustained in only some vascular endothelial cells.

Fluorescence immunostaining was performed using antibodies against CD31 (red) and CD157 (green), with DAPI (blue) used for nuclear staining. As a result, almost all cells were positive for CD31. For CD157, most cells were positive on day 14 but the number of positive cells decreased by day 24 (Fig. 2c). This result was consistent with the findings from qRT-PCR and flow cytometric analyses showing that CD157 expression on endothelial cells gradually decreased at transcriptional and cell surface protein levels, respectively.

### High expression of ABC transporters in CD157^+^ cells

**Fig. 3:**
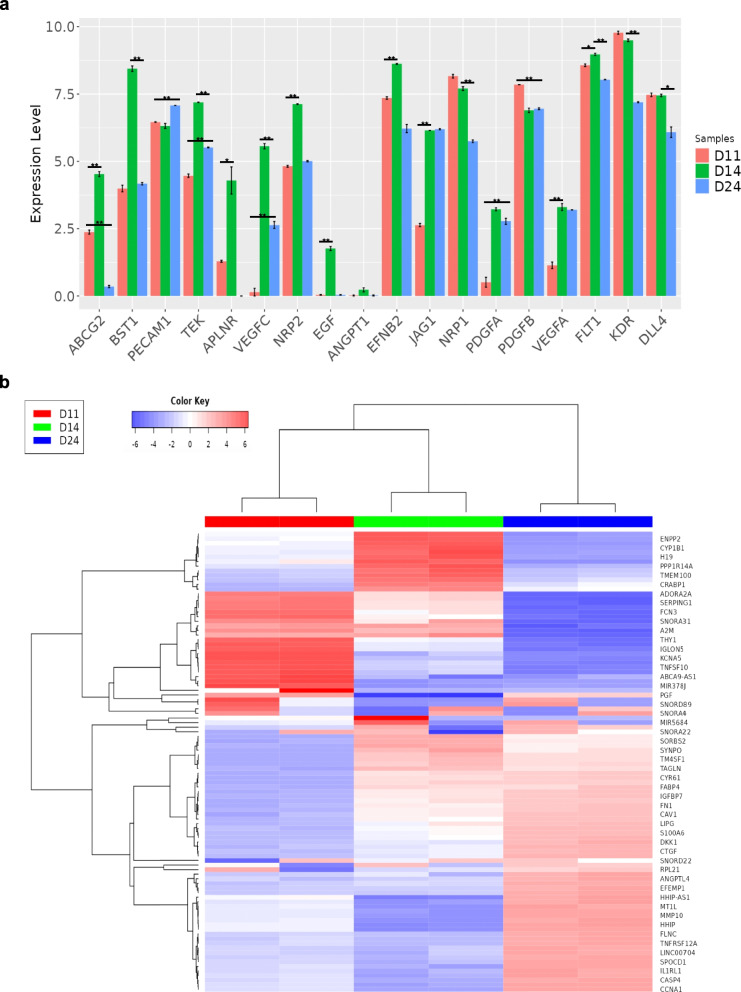
Comprehensive gene expression analysis using RNA sequencing. **a** Changes in expression of angiogenesis-related genes. (*P < 0.05, **P < 0.01) on days 11, 14, and 24. **b** Heatmap of the top 100 genes on days 11 (CD157^−^), 14 (CD157^+^), and 24 (CD157.^−^)

Hoechst analysis is a method of distinguishing stem cells from somatic cells based on the principle that stem cells with drug efflux capabilities can expel Hoechst blue and Hoechst red dyes. The process of drug efflux is known to rely on the activity of ATP-binding cassette (ABC) transporters. Therefore, we conducted qRT-PCR analysis of the relative gene expression of *ABCG2*, a representative ABC transporter, on days 11, 14 (enriched in CD157^+^ cells), and 24 (depleted in CD157^+^ cells). A significantly higher expression of *ABCG2* was noted on day 14 suggesting that CD157^+^ endothelial cells were resistant to toxic agent through their expression of ABC transporters in human CD157^+^ cells (Fig. 3a). In this analysis, we also showed that endothelial cells appeared, on day 14, to highly express CD157 (BST-1) and other vascular endothelial cell–specific gene signatures, i.e., *PECAM1* (*CD31*), *TEK* (*Tie2*), and *APJ* (*APLNR*), as well as others.

### Comprehensive gene analysis in CD157^−^ and CD157^+^ cell populations

Previous studies have demonstrated that endothelial cells expressing CD157 in mice exhibit high colony-forming and angiogenic capabilities. In this study, we performed RNA sequencing analysis using cells from days 11, 14, and 24 to comprehensively investigate gene expression in human CD157^+^ and CD157^−^ endothelial cells. The heatmap of the top 100 genes revealed a significant increase in genes such as *ENPP2*, encoding autotaxin, and *CYP1B1*, encoding one of the cytochrome P450 enzymes, on day 14 (Fig. 3b). When comparing the expression of genes related to angiogenesis only a few genes were decreased specifically in CD157^+^ cells while genes such as *Tie2* (*TEK*), *APJ* (*APLNR*), *VEGFC*, *NRP2*, and *EGF* were markedly increased.

**Fig. 4:**
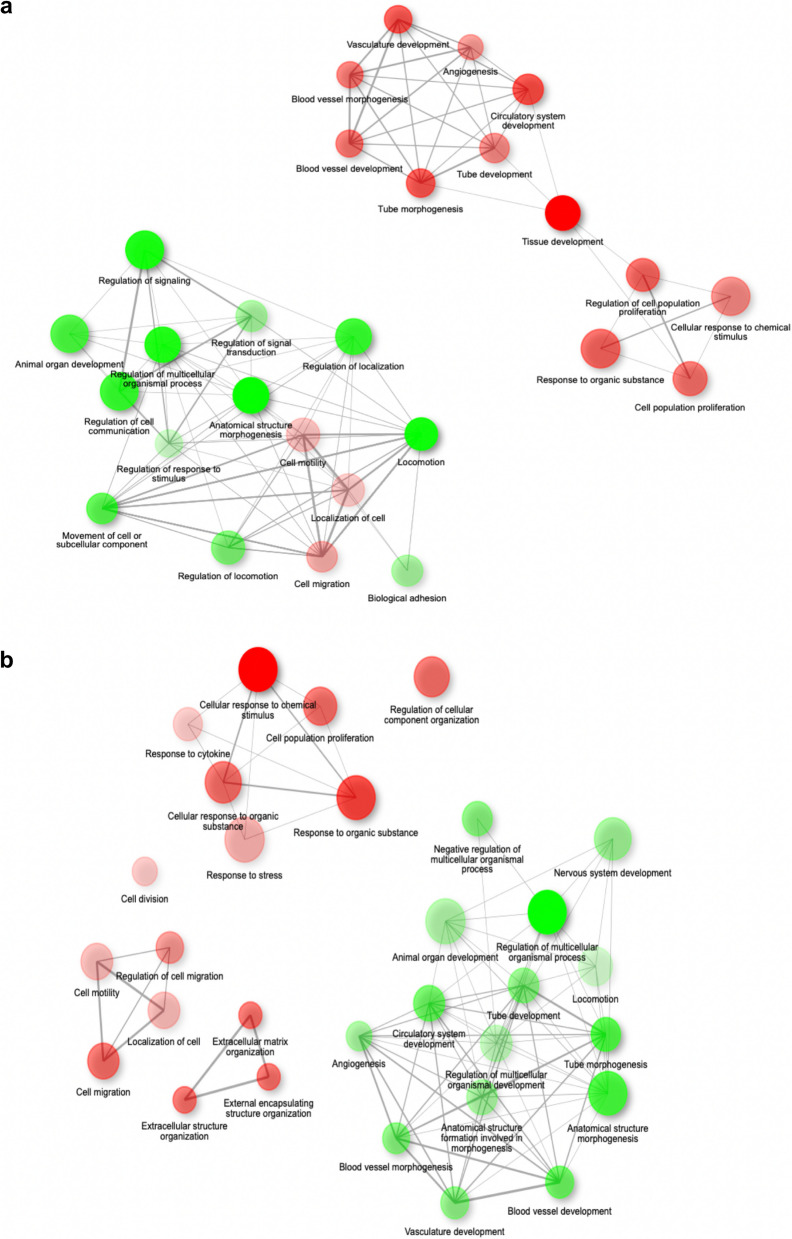
Network image from enrichment analysis. **a** Network image of differentially expressed gene (DEG) enrichment analysis comparing days 11 and 14. Red and green indicate gene expression groups that are increased and decreased, respectively, on day 14. **b** Network image of day 14 compared to day 24. Red and green indicate gene expression groups that are increased and decreased on day 24, respectively

Enrichment analysis of differentially expressed genes (DEGs) using a Gene Ontology Biological Process was visualized in network images. When comparing day 11 (predominantly CD157^−^ population) and day 14 (predominantly CD157^+^ population), an increase in gene groups related to blood vessel morphogenesis, angiogenesis, blood vessel development, tube morphogenesis, and cell population proliferation was observed on day 14 (Fig. 4a). In comparison, comparing day 14 (predominantly CD157^+^ population) with day 24 (predominantly CD157^−^ population) revealed a decrease in the expression of gene groups related to blood vessel morphogenesis, angiogenesis, blood vessel development, and tube morphogenesis on day 24 (Fig. 4b). These results suggested that CD157^+^ cells represent a more undifferentiated cell population and may be associated with cells involved in angiogenesis and vascular morphology.

### Tube formation assay in CD157^−^ and CD157^+^ cell populations

**Fig. 5:**
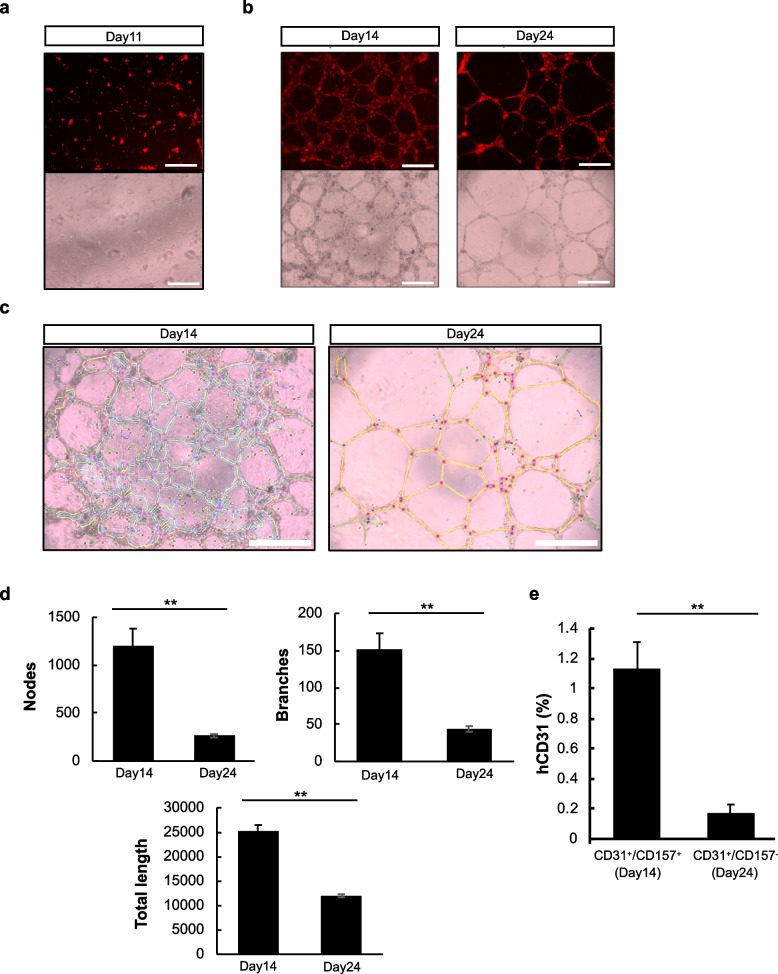
Tube formation assay and transplantation using CD31⁺ CD157^+^ and CD31⁺ CD157^−^ cell populations. **a** Tube formation assay using endothelial cells on day 11. Red indicates acetylated low density lipoprotein (Alexa Fluor™ 594-Ac-LDL) uptake by endothelial cells. **b** Tube formation assay using endothelial cells from days 14 and 24. Red indicates Alexa Fluor™ 594-Ac-LDL. scale bar = 500 μm. **c** Image J Angiogenesis Analyzer results of tube formation assay observed in **b**. **d** Quantification, by ImageJ Angiogenesis Analyzer, of nodes, branches, and total length of endothelial cells (**P < 0.01). **e** Percentage of human CD31^+^ cells in hind limbs after transplantation of CD31^+^/CD157^+^ endothelial cells isolated from a day 14 culture or CD31^+^/CD157.^−^ endothelial cells isolated from a day 24 culture. *n* = 3. (**P < 0.01)

Gene expression analysis from RNA sequencing revealed an upregulation of genes associated with tube morphogenesis in CD157^+^ cells. Based on this result, a tube formation assay was conducted to investigate whether the presence or absence of CD157 expression in iPSC-ECs could influence tube formation. No cell aggregation was observed on day 11, when CD157 expression had not yet occurred, suggesting an immature state of endothelial cells. On day 14, with a sharp increase in CD157 expression, and on day 24, when CD157 expression then decreased, tube-like structures were observed. However, significant morphological differences were evident and CD157^+^ cells formed relatively thicker tubes (Fig. 5a, b). Furthermore, quantitative analysis using ImageJ to measure nodes, branches, and total length per unit area revealed significantly higher values on day 14 across all parameters, indicating enhanced tube formation capabilities (Fig. 5c, d).

**Fig. 6:**
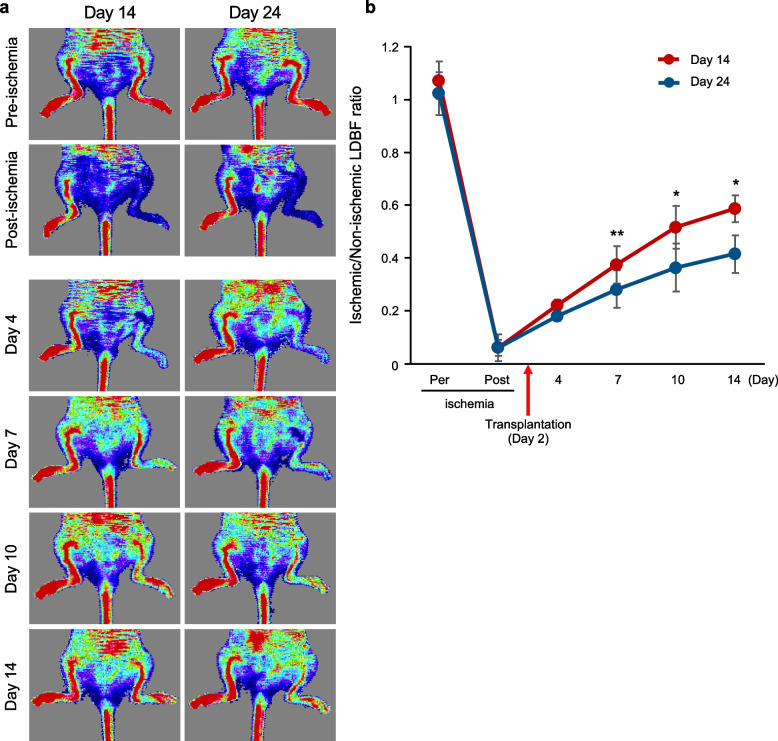
Transplantation of day 14 iPSC-ECs improved lower limb ischemia more effectively than day 24 iPSC-ECs. Representative LDBF images (**a**) and a line graph of the calculated LDBF ratio (**b**) are shown. **a** Blood flow is visualized as changes in laser Doppler frequency using pseudocolor imaging. A significant increase in blood flow was observed in the group transplanted with day 14 iPSC-ECs compared to the day 24 group. **b** Quantitative analysis of the ischemic/non-ischemic LDBF ratio in mice transplanted with day 14 iPSC-ECs (red) or day 24 iPSC-ECs (blue). *n* = 4 per group. (*P < 0.05, **P < 0.01)

Next, we observed in vivo engraftment by transplanted iPSC-ECs. Without any preconditioning, endothelial cells cannot be incorporated in mice. Therefore, we induced hind limb ischemia by occlusion of the femoral artery. CD157^+^ or CD157^−^ vascular endothelial cells on days 14 or 24, respectively, of iPSC culture as above were inoculated into hind limb muscles. After 2 weeks of transplantation, cells from hind limbs were analyzed by flow cytometry. As indicated in Fig. 5e, in hind limb muscles with transplanted CD157^+^ endothelial cells more human endothelial cells stained with human CD31 antibody compared to in hind limb muscles with transplanted CD157^−^ endothelial cells. We also confirmed that human CD31^+^ endothelial cells derived from CD157^+^ but not CD157^−^ iPSC-ECs were incorporated in hind limb muscle of this mouse model (Supplementary Fig. S1). Moreover, we found that transplantation of day 14 iPSC-ECs improved lower limb ischemia more effectively than that of day 24 iPSC-ECs (Fig. 6). These indicated that a CD157^+^ endothelial cell population has a higher ability to repopulate in vivo.

## Discussion

We found that immature endothelial cells (CD31^+^/CD157^−^) appeared on day 9 during the process of human iPSCs differentiating into vascular endothelial cells, followed by the appearance of CD157^+^ cells from day 14, and further followed by a decrease in CD157^+^ cells. These results are consistent with the results of single-cell analysis using vascular endothelial cells from a developmental stage for postnatal and adult mice in vivo [15]. This suggested that in humans, as in mice, *CD157* is not a gene expressed during vascular formation in early development but is expressed during postnatal angiogenesis.

In mice, CD157^+^ endothelial cells emerge at around birth and exist for their entire life span. Unlike the in vivo results in mice, most human iPSC–derived endothelial cells became negative for CD157 soon after their emergence. The reason for this may be attributed to the difference between an in vitro culture environment and actual conditions in a human body. For example, the oxygen concentration in the uterus is said to be from 2 to 8%, which differs significantly from the oxygen concentration in air (20.9%). This is of interest considering that several papers have shown that stemness is maintained in hypoxic environments [16, 17]. Besides oxygen status, growth factors may also alter the behavior of CD157^+^ endothelial cells. Further investigation is required to identify how the stemness of endothelial cells is maintained in a so-called microenvironmental niche.

We obtained results showing that about 10% of the CD157^+^ cell population was maintained after day 24 of culture; however, this was when endothelial cells did not proliferate. This indicates that CD157 is expressed on endothelial cells in culture; however, such cells are thought to have already started aging, with cell division being inhibited. We previously reported that CD157^+^ cells from aged mice not only have an impaired ability to form in vitro endothelial cell colonies, but also showed a reduced ability to construct blood vessels during transplantation [18]. Therefore, we considered that CD157^+^ endothelial cells derived from human iPSCs after day 24 in this study also had impaired stemness, regardless of their CD157 positive status.

RNA sequencing analysis comparing CD157^−^ and CD157^+^ cells showed significantly higher expression of *ENPP2*, which encodes autotaxin (*ATX*), and *CYP1B1*, a cytochrome P450 gene, in CD157⁺ cells. The *ATX* gene induces cell migration, metastasis, and angiogenesis by producing lysophosphatidic acid, a motility factor [19]. It has also been reported that *CYP1B1* has a significant effect on angiogenesis and inflammatory function through the generation of endothelial nitric-oxide synthase [20]. In CYP1B1^−/−^ liver sinusoidal endothelial cells, the expression levels of VEGF, BMP6, VE cadherin, and ZO-1 are decreased, indicating reduced angiogenic potential and maturation of vascular structure. Therefore, we hypothesized that such genes may be involved in the increased angiogenic potential of CD157 observed in this study.

Furthermore, RNA sequencing analysis revealed that the angiogenesis-related genes, *Tie2* (*TEK*) and *APJ* (*APLNR*), are upregulated in CD157^+^ cells. Tie2 is a tyrosine kinase receptor for angiopoietin-1 (Ang1) or Ang2. Tie2 has been shown to be activated by Ang1 secreted from pericytes and hematopoietic stem cells, resulting in activation of Erk and Akt and, consequently, in the induction of angiogenesis and vascular stabilization [21, 22]. Therefore, it is suggested that Tie2 expression may be upregulated in human CD157^+^ cells derived from iPSCs, which may increase their angiogenic potential and the formation of structurally stable larger blood vessels as observed in Fig. 5b.

The apelin receptor (*APJ*), a G protein–coupled receptor with seven transmembrane domains, is also a gene involved in angiogenesis, especially in the maturation process of blood vessel formation. Apelin is an endogenous APJ receptor agonist and is involved in many physiological functions in vivo, including vasodilation, angiogenesis, skeletal muscle regeneration, and the enhancement of cardiac contractility [23]. We previously showed that Ang1 induces apelin secretion from vascular endothelial cells through Tie2–Ang1 signaling and that apelin activates APJ by an autocrine loop, thereby increasing the lumen diameter of blood vessels [14, 24]. In the present study, CD157^+^ cells showed a larger lumen diameter than CD157^−^ cells in tube formation; we hypothesize that activation of APJ in CD157^+^ cells is involved in this process. It has also been shown that APJ expression is higher in vascular endothelial cells isolated from lung cancer tissue that exhibits undifferentiated characteristics [25]. In future, we would like to determine whether Tie2 and APJ are actually involved in the maintenance of stemness and the increased angiogenic potential of CD157^+^ cells.

By utilizing a human iPSC–based vascular endothelial cell differentiation system and comprehensive genetic analysis using RNA sequencing, our study shows that CD157^+^ cells exhibit elevated levels of genes associated with vascular morphology, angiogenesis, and tube formation. These results suggest that human CD157^+^ cells, like those in mice, function as endothelial stem-like cells. However, how stemness and angiogenic potential are induced in CD157^+^ endothelial cells remains to be elucidated. Therefore, we aim to study the function of genes observed in CD157^+^ endothelial cells in order to analyze the stemness of endothelial cells in future. The differences in gene expression between CD157^+^ endothelial cells derived from iPSCs and those present in vivo need to be thoroughly investigated. The findings from this study suggest that CD157^+^ cells could be utilized for vascular regeneration therapy; however, CD157^+^ endothelial cells collected from humans are considered to be safer and more economically advantageous than endothelial cells derived from iPSCs.

## Conclusion

CD157^+^ vascular endothelial cells derived from human iPSCs show higher expression of genes contributing to angiogenesis and have higher angiogenic potential than CD157^−^ vascular endothelial cells.

## Supplementary Information

Supplementary Material 1.

Supplementary Material 2.

## Data Availability

RNA-seq data are available at the Gene Expression Omnibus (GEO) under accession number GSExxxxxxx (We are currently registering our data in GEO; an accession number will be provided at the time of publication).

## Abbreviations

ABC: ATP-binding cassette
Ac-LDL: Acetylated low density lipoprotein
Ang: Angiopoietin
APJ: Apelin receptor
ATX: Autotaxin
DAPI: 4″,6-Diamino-2-phenylindole
DEGs: Differentially expressed genes
FPKM: Fragments per kilobase of exon per million mapped fragments
HUVECs: Human umbilical vein endothelial cells
iPSCs: Induced pluripotent stem cells
iPSC-ECs: Endothelial cells derived from iPSCs
LDBF: Laser Doppler Blood Flow
PBS: Phosphate-buffered saline
PBST: PBS with 0.5% Tween-20
qRT-PCR: Quantitative reverse transcription-PCR
SP: Side population
VEGFR: Vascular endothelial growth factor receptor
