# Supplementary material for: CD157+ vascular endothelial cells derived from human-induced pluripotent stem cells have high angiogenic potential
Source: Inflamm Regen. 2025 May 14;45:14. doi: 10.1186/s41232-025-00379-0 (PMC12077006; doi:10.1186/s41232-025-00379-0)
Supplement: Supplementary file 1 — Supplementary Material 1. [file 41232_2025_379_MOESM1_ESM.pdf]

Table1. PCR Primers used in the experiment.

Primer name	Forward Primer 5'→3'	Reverse Primer5'→3'
**GAPDH**	CCACCCATGGCAAATTCC	TGGGATTTCCATTGATGACAAG
**CD31**	AGACGTGCAGTACACGGAAG	AGGGACAGCTTTCCGGACTT
**CD157**	AATGGTTCAGAGCCAACAGG	CCCCAATTTCATGCATAACC
**OCT3/4**	AAGCAGAAACCCTCGTGCAG	CACCGCAGGAACAAATTCTCC
**SOX2**	ATGGGAGGGGTGCAAAAGAG	GCTGTCATTTGCTGTGGGTG
**VEGFR2**	AGGGGAACTGAAGACAGGCTA	CTGGCATCATAAGGCAGTCGT
**VEGFR1**	GCTTTGAAACTTGCCTGGGG	GCCCACTTGATCTTTAGACCCT
**TBXT**	CCACCAGTCCTACTTTAGTGAGA	ACCTGCTGTCCTCAACTATGA
**ABCG2**	GAGCCTACAACTGGCTTAGACTCAA	TGATTGTTCGTCCCTGCTTAGAC
