# Supplementary material for: CD157+ vascular endothelial cells derived from human-induced pluripotent stem cells have high angiogenic potential
Source: Inflamm Regen. 2025 May 14;45:14. doi: 10.1186/s41232-025-00379-0 (PMC12077006; doi:10.1186/s41232-025-00379-0)
Supplement: Supplementary file 2 — Supplementary Material 2. [file 41232_2025_379_MOESM2_ESM.pdf]

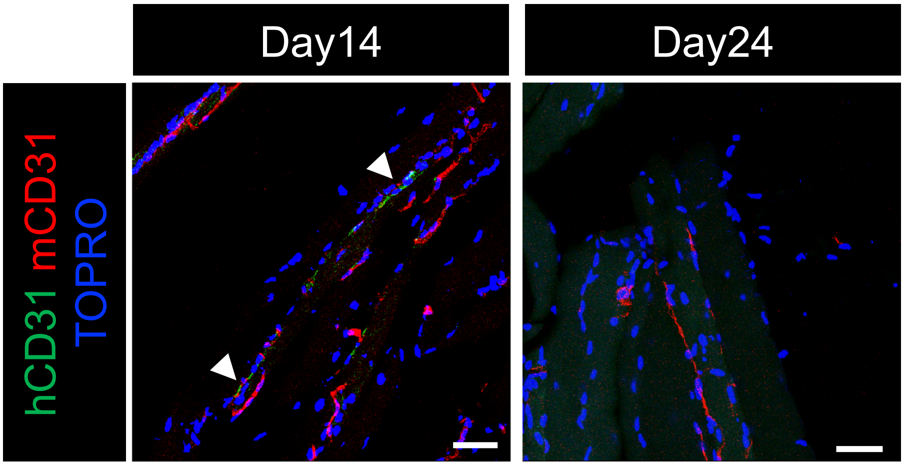

Figure S1. Histological analysis of hind limb muscles. Muscle sections transplanted with Day 14 iPSC-ECs or Day24 iPSC-ECs were stained with anti-human (h) CD31 or mouse (m) CD31 antibodies. Nuclei were counterstained with TOPRO. Arrow heads indicate human ECs. Scale bar; 50μm.
